# Supplementary material for: Ambient ozone pollution at a coal chemical industry city in the border of Loess Plateau and Mu Us Desert: characteristics, sensitivity analysis and control strategies
Source: PeerJ. 2021 Apr 27;9:e11322. doi: 10.7717/peerj.11322 (PMC8086580; doi:10.7717/peerj.11322)
Supplement: Supplemental Information 1 — Table S1 Summary of GC-MS/FID method for target compounds and their concentrations, Table S2 Over-standard rate of ozone concentration in different ranges of temperature, relative humidity, wind speed and wind direction, Table S3 Average trace gas concentrations and meteorological parameters during six O3 episode periods and VOCs sampling period, Fig. S1 (A) Monthly variation of MDA8-90th O3 concentrations and (B) diurnal variation of 1-hour average O3 concentrations for four sites, Fig. S2 48 h backward trajectory on 26 July (A), 1 (B) and 10 (C) August, Fig. S3 PMF source profile. [file peerj-09-11322-s001.docx]

**Ambient ozone pollution at a coal chemical industry city in the border of Loess Plateau and Mu Us Desert: Characteristics, sensitivity analysis and control strategies**

Manfei Yin^1,2^, Xin Zhang^2,3^, Yunfeng Li^2,3^, Kai Fan^4^, Hong Li^2^, Rui Gao^2*^, Jinjuan Li^1*^

^1.^ College of Resource and Environment Engineering, Guizhou University, Guiyang, China

^2.^ State Key Laboratory of Environmental Criteria and Risk Assessment, Chinese Research Academy of Environmental Sciences, Beijing, China

^3.^ Environment Research Institute, Shandong University, Qingdao, China

^4.^ Yulin Municipal Ecology and Environment Bureau, Yulin, China

Corresponding Author:

Rui Gao^2^

No. 8 Dayangfang Street, Chaoyang District, Beijing, 100055, China

Email address: gaorui@craes.org.cn

Jinjuan Li^1^

No. 2708, Huaxi Dadao Street, Guiyang, 550025, China

Email address: summy_lee@163.com

Table S1 Summary of GC-MS/FID method for target compounds and their concentrations

| **VOCs specie** | **Mean concentration ( ppbv)** | **Standard deviation ( ppbv)** | **Calibration R^2 *^** | **RSD^**^** |
| --- | --- | --- | --- | --- |
| **Ethane** | 5.43 | 2.99 | 0.999846 | 0.53% |
| **Propane** | 1.99 | 2.52 | 0.999998 | 0.55% |
| **Iso-butane** | 1.18 | 0.75 | 0.999988 | 0.99% |
| **n-Butane** | 1.27 | 1.04 | 0.999992 | 0.61% |
| **Iso-pentane** | 1.23 | 0.74 | 0.999926 | 1.15% |
| **n-Pentane** | 0.44 | 0.52 | 0.991416 | 1.20% |
| **2,2-Dimethylbutane** | 0.03 | 0.01 | 0.999838 | 2.72% |
| **2,3-Dimethylbutane** | 0.12 | 0.11 | 0.99993 | 1.10% |
| **2-Methyl pentane** | 0.13 | 0.11 | 0.999952 | 1.04% |
| **3-Methyl pentane** | 0.06 | 0.05 | 0.999934 | 0.88% |
| **n-Hexane** | 0.14 | 0.12 | 0.999932 | 1.04% |
| **2-Methyl hexane** | 0.03 | 0.02 | 0.999948 | 1.05% |
| **3-Methyl hexane** | 0.03 | 0.02 | 0.99997 | 1.11% |
| **n-Heptane** | 0.06 | 0.03 | 0.999934 | 1.07% |
| **n-Octane** | 0.22 | 0.02 | 0.999968 | 0.91% |
| **n-Nonane** | 0.02 | 0.01 | 0.999878 | 1.31% |
| **n-Decane** | 0.01 | 0.00 | 0.999906 | 1.15% |
| **n-Undecane** | 0.01 | 0.00 | 0.99978 | 1.32% |
| **Ethene** | 1.22 | 1.17 | 0.999996 | 0.55% |
| **Propene** | 0.07 | 0.20 | 0.999656 | 2.21% |
| **Trans-2-butene** | 0.01 | 0.04 | 0.99999 | 0.56% |
| **Cis-2-butene** | 0.02 | 0.07 | 0.999722 | 1.84% |
| **1-Butene** | 0.85 | 0.78 | 0.999882 | 4.90% |
| **1,3-Butadiene** | 0.01 | 0.01 | 0.999956 | 1.22% |
| **1-Pentene** | 0.17 | 0.18 | 0.999934 | 1.18% |
| **Trans-2-pentene** | 0.02 | 0.02 | 0.999962 | 1.22% |
| **Isoprene** | 0.10 | 0.12 | 0.99996 | 1.13% |
| **Cis-2-pentene** | 0.08 | 0.06 | 0.99998 | 0.83% |
| **1-Hexene** | 0.25 | 0.09 | 0.999958 | 1.08% |
| **Ethyne** | 1.18 | 2.14 | 0.999988 | 3.76% |
| **Methyl bromide** | 0.01 | 0.00 | 0.999914 | 1.19% |
| **Ethyl chloride** | 0.00 | 0.01 | 0.999916 | 1.17% |
| **1,1-Dichloroethene** | 0.00 | 0.00 | 0.999908 | 1.09% |
| **Dichloromethane** | 0.29 | 0.46 | 0.999924 | 0.90% |
| **1,1-Dichloroethane** | 0.02 | 0.03 | 0.999926 | 1.07% |
| **Cis-1,2-dichloroethene** | 0.02 | 0.03 | 0.999928 | 0.93% |
| **Chloroform** | 0.17 | 0.31 | 0.999928 | 1.11% |
| **1,2-Dichloroethane** | 0.00 | 0.00 | 0.999856 | 1.09% |
| **Trichloroethylene** | 0.11 | 0.19 | 0.999928 | 1.17% |
| **1,2-Dichloropropane** | 0.01 | 0.01 | 0.999906 | 1.18% |
| **1,1,2-Trichloroethane** | 0.13 | 0.10 | 0.999848 | 1.07% |
| **Perchloroethylene** | 0.01 | 0.05 | 0.99986 | 1.18% |
| **p-Dichlorobenzene** | 0.26 | 0.45 | 0.9999 | 1.44% |
| **o-Dichlorobenzene** | 0.00 | 0.00 | 0.999898 | 1.41% |
| **Benzene** | 0.01 | 0.00 | 0.99991 | 1.14% |
| **Toluene** | 0.00 | 0.00 | 0.99981 | 1.07% |
| **Ethyl benzene** | 0.64 | 0.24 | 0.99988 | 1.15% |
| **p-Xylene** | 0.47 | 0.36 | 0.999214 | 1.16% |
| **o-Xylene** | 0.15 | 0.10 | 0.999874 | 1.29% |
| **Styrene** | 0.42 | 0.33 | 0.999746 | 1.28% |
| **Iso-propyl benzene** | 0.12 | 0.09 | 0.99989 | 1.22% |
| **n-Propyl benzene** | 0.06 | 0.06 | 0.999786 | 1.10% |
| **m-Ethyl toluene** | 0.01 | 0.02 | 0.999856 | 1.05% |
| **p-Ethyl toluene** | 0.01 | 0.00 | 0.998688 | 1.09% |
| **1,3,5-Trimethyl benzene** | 0.03 | 0.02 | 0.99908 | 1.13% |
| **o-Ethyl toluene** | 0.01 | 0.01 | 0.99981 | 1.14% |
| **1,2,4-Trimethyl benzene** | 0.01 | 0.01 | 0.999894 | 1.21% |
| **1,2,3-Trimethyl benzene** | 0.01 | 0.01 | 0.999896 | 1.19% |
| **Ethanol** | 0.04 | 0.03 | 0.999952 | 1.12% |
| **Acrolein** | 0.01 | 0.01 | 0.999954 | 0.97% |
| **Acetone** | 3.14 | 2.24 | 0.998876 | 3.70% |
| **Isopropyl alcohol** | 0.02 | 0.01 | 0.999708 | 6.40% |
| **Methyl t-butyl ether** | 0.50 | 0.20 | 0.999964 | 0.98% |
| **Methyl ethyl ketone** | 0.15 | 0.08 | 0.99998 | 1.03% |
| **Ethyl acetate** | 0.14 | 0.13 | 0.999726 | 1.66% |
| **4-Methyl-2-entanone** | 0.09 | 0.04 | 0.999952 | 0.98% |
| **2-Hexanone** | 0.24 | 0.07 | 0.99937 | 1.19% |
| * R^2^ greater than 0.99 is considered as qualified. | | | | |
| ** RSD: Relative standard deviation of each VOCs specie concentration. RSD greater than 10% is considered as qualified | | | | |

Table S2 Average temperature, relative humidity and wind speed in April and September from 2017-2019.

| **Year** | **Month** | **Temperature (℃)** | **Relative humidity (%)** | **Wind speed (m/s)** |
| --- | --- | --- | --- | --- |
| **2017** | April | 13±5 | 42±26 | 1.8±1.2 |
|  | September | 20±4 | 61±20 | 1.6±0.9 |
| **2018** | April | 15±7 | 39±20 | 1.9±1.2 |
|  | September | 15±4 | 67±23 | 1.5±0.9 |
| **2019** | April | 15±4 | 42±21 | 1.7±0.9 |
|  | September | 19±5 | 65±21 | 1.4±0.9 |
| **Mean** | April | 14±6 | 41±24 | 1.8±1.2 |
|  | September | 18±5 | 64±21 | 1.5±0.9 |

Table S3 Average trace gas concentrations and meteorological parameters during six O_3_ episode periods and VOCs sampling period.

|  |  | **T**  **( ℃)** | **RH**  **( %)** | **WS**  **( m/s)** | **NO_2_**  **( ppbv)** | **CO**  **( ppmv)** | **O_3_**  **( ppbv)** | **PM_2.5_**  **( ppbv)** | **PM_10_**  **( ppbv)** | **TVOC**  **( ppbv)** | **MDA8**  **( ppbv)** |
| --- | --- | --- | --- | --- | --- | --- | --- | --- | --- | --- | --- |
| **Sampling period**  **(7 July-10 August)** | **Mean** | 24.0 | 64.3 | 1.6 | 12.9 | 0.7 | 49.5 | 19.6 | 40.0 | 26.1 | 71.5 |
|  | **Standard deviation** | 4.1 | 18.9 | 0.8 | 8.3 | 0.2 | 22.0 | 8.7 | 19.3 | 12.4 | 8.9 |
|  | **Maximum** | 33.9 | 100.0 | 4.5 | 49.7 | 1.4 | 102.2 | 56.0 | 98.0 | 90.4 | 85.1 |
|  | **Minimum** | 15.1 | 14.0 | 0.2 | 2.9 | 0.3 | 0.5 | 1.0 | 2.0 | 5.8 | 50.3 |
| **O3 episode period Ⅰ**  **(11-14 July)** | **Mean** | 24.9 | 51.3 | 1.2 | 16.7 | 0.7 | 54.0 | 20.4 | 42.1 | 29.9 | 82.9 |
|  | **Standard deviation** | 6.1 | 18.6 | 0.6 | 11.2 | 0.2 | 27.7 | 8.6 | 18.4 | 12.0 | 2.4 |
|  | **Maximum** | 32.9 | 81.0 | 4.1 | 47.2 | 1.2 | 102.2 | 40.0 | 88.0 | 58.4 | 85.1 |
|  | **Minimum** | 17.9 | 25.0 | 0.3 | 5.8 | 0.3 | 2.3 | 10.0 | 18.0 | 13.3 | 79.6 |
| **O3 episode period Ⅱ**  **(20 July)** | **Mean** | 26.2 | 61.2 | 1.0 | 18.5 | 0.7 | 38.3 | 18.8 | 51.1 | 41.4 | 75.2 |
|  | **Standard deviation** | 4.4 | 21.2 | 0.5 | 8.6 | 0.3 | 33.0 | 7.7 | 16.7 | 23.5 |  |
|  | **Maximum** | 32.1 | 89.0 | 1.8 | 33.1 | 1.4 | 79.8 | 37.0 | 90.0 | 90.4 |  |
|  | **Minimum** | 20.4 | 35.0 | 0.2 | 5.8 | 0.5 | 0.5 | 8.0 | 34.0 | 14.4 |  |
| **O3 episode period Ⅲ**  **(25-26 July)** | **Mean** | 27.2 | 52.5 | 2.0 | 13.9 | 0.6 | 53.9 | 21.2 | 51.2 | 27.5 | 81.9 |
|  | **Standard deviation** | 4.3 | 16.3 | 1.1 | 8.9 | 0.3 | 31.0 | 7.0 | 19.0 | 12.3 | 3.0 |
|  | **Maximum** | 33.2 | 84.0 | 4.5 | 37.0 | 1.4 | 89.6 | 32.0 | 86.0 | 51.4 | 84.1 |
|  | **Minimum** | 18.9 | 26.0 | 0.3 | 4.4 | 0.3 | 0.9 | 11.0 | 31.0 | 13.1 | 79.8 |
| **O3 episode period Ⅳ**  **(28 July)** | **Mean** | 27.2 | 68.6 | 1.8 | 10.2 | 0.6 | 54.3 | 22.3 | 52.2 | 26.6 | 77.2 |
|  | **Standard deviation** | 4.2 | 16.3 | 0.9 | 6.0 | 0.1 | 20.8 | 5.7 | 23.3 | 9.0 |  |
|  | **Maximum** | 33.9 | 97.0 | 3.4 | 25.3 | 0.8 | 88.2 | 33.0 | 94.0 | 42.1 |  |
|  | **Minimum** | 19.2 | 44.0 | 0.7 | 3.9 | 0.5 | 21.5 | 12.0 | 21.0 | 16.0 |  |
| **O3 episode period Ⅴ**  **(30 July-2 August)** | **Mean** | 25.2 | 55.7 | 1.7 | 15.3 | 0.6 | 52.1 | 23.9 | 48.9 | 31.0 | 78.9 |
|  | **Standard deviation** | 3.7 | 14.4 | 0.9 | 9.9 | 0.2 | 26.0 | 8.4 | 20.9 | 18.1 | 3.4 |
|  | **Maximum** | 32.8 | 81.0 | 4.4 | 42.4 | 1.0 | 89.1 | 47.0 | 98.0 | 59.1 | 82.1 |
|  | **Minimum** | 19.5 | 21.0 | 0.4 | 3.9 | 0.3 | 0.9 | 10.0 | 15.0 | 11.5 | 74.8 |
| **O3 episode period Ⅵ**  **(10 August)** | **Mean** | 23.3 | 61.9 | 1.4 | 12.0 | 0.6 | 59.1 | 21.2 | 45.0 | 13.0 | 84.1 |
|  | **Standard deviation** | 4.1 | 13.1 | 0.5 | 5.7 | 0.1 | 20.0 | 9.4 | 17.2 | 4.6 |  |
|  | **Maximum** | 28.8 | 83.0 | 2.4 | 25.3 | 0.8 | 96.1 | 46.0 | 69.0 | 20.8 |  |
|  | **Minimum** | 17.4 | 43.0 | 0.4 | 5.4 | 0.5 | 32.7 | 7.0 | 13.0 | 5.8 |  |
| TVOCs: Total Volatile organic compounds. | | | | | | | | | | | |

Table S4 Cities with over standard O_3_ concentration around Yulin City during EP3, EP4 and EP5.

|  | **Date** | **Cities of MDA8 O_3_ over 75 ppbv** |
| --- | --- | --- |
| **EP3** | 2019/7/23 | Linfen |
|  | 2019/7/24 | Linfen |
|  | 2019/7/25 | Yuncheng; Linfen; Weinan |
|  | 2019/7/26 | Yuncheng; Linfen; Weinan |
| **EP4** | 2019/7/26 | Yuncheng; Linfen; Weinan |
|  | 2019/7/27 | Linfen; Weinan; Xianyang; Xi'an |
|  | 2019/7/28 | Linfen |
| **EP5** | 2019/7/30 | Linfen; Lyuliang |
|  | 2019/7/31 | Linfen; Yuncheng; Jiaozuo; Jincheng |
|  | 2019/8/1 | Linfen |
|  | 2019/8/2 | Linfen; Lyuliang |

Table S5 Over-standard rate of ozone concentration in different ranges of temperature, relative humidity, wind speed and wind direction.

| **Meteorological**  **factors** | **Group** | **Total days** | **MDA1**  **polluted days** | **MDA8 polluted days** | **MDA1 polluted days ratio** | **MDA8 polluted days ratio** |
| --- | --- | --- | --- | --- | --- | --- |
| **Temperature**  **(T)** | 10-15 | 89 | 0 | 0 | 0.00% | 0.00% |
|  | 15-20 | 278 | 0 | 3 | 0.00% | 1.08% |
|  | 20-25 | 728 | 39 | 145 | 5.36% | 19.92% |
|  | 25-30 | 369 | 33 | 159 | 8.94% | 43.09% |
|  | >30 | 11 | 1 | 9 | 9.09% | 81.82% |
| **Relative humidity**  **(RH)** | 20-40 | 314 | 19 | 71 | 6.05% | 22.61% |
|  | 40-60 | 479 | 43 | 150 | 8.98% | 31.32% |
|  | 60-80 | 482 | 11 | 93 | 2.28% | 19.29% |
|  | 80-100 | 193 | 0 | 2 | 0.00% | 1.04% |
| **Wind speed**  **(WS)** | 0-1.5 | 1025 | 59 | 231 | 5.76% | 22.54% |
|  | 1.5-3 | 393 | 13 | 80 | 3.31% | 20.36% |
|  | 3-4.5 | 54 | 1 | 5 | 1.85% | 9.26% |
|  | >4.5 | 3 | 0 | 0 | 0.00% | 0.00% |
| **Wind direction**  **(WD)** | North | 296 | 15 | 33 | 5.07% | 11.15% |
|  | East | 325 | 12 | 91 | 3.69% | 28.00% |
|  | South | 488 | 28 | 129 | 5.74% | 26.43% |
|  | West | 366 | 21 | 63 | 5.74% | 17.21% |





Figure S1 CO, PM_2.5_, PM_10_, NO_2_ and O_3_ concentrations in 2017 to 2019 (except for the unit of CO is ppmv, other pollutants are all ppbv).


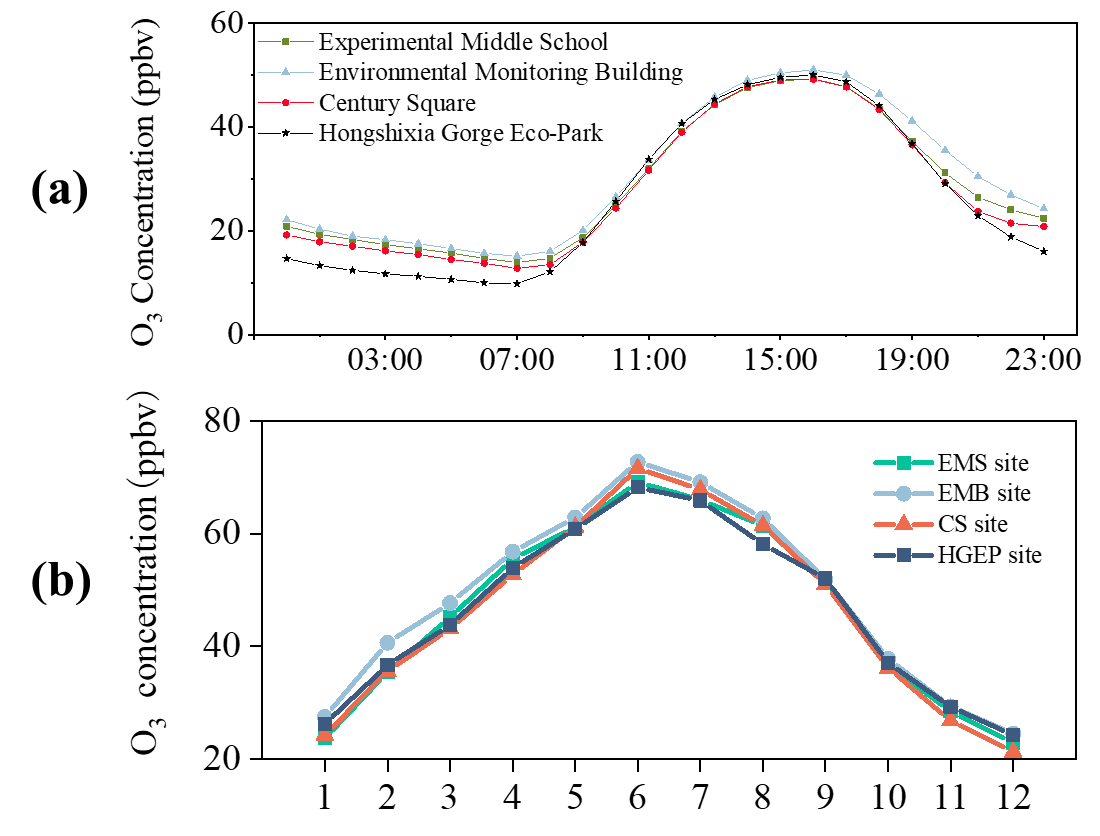


Figure S2 (a) Monthly variation of MDA8-90th O_3_ concentrations and (b) diurnal variation of 1-hour average O_3_ concentrations for four sites.


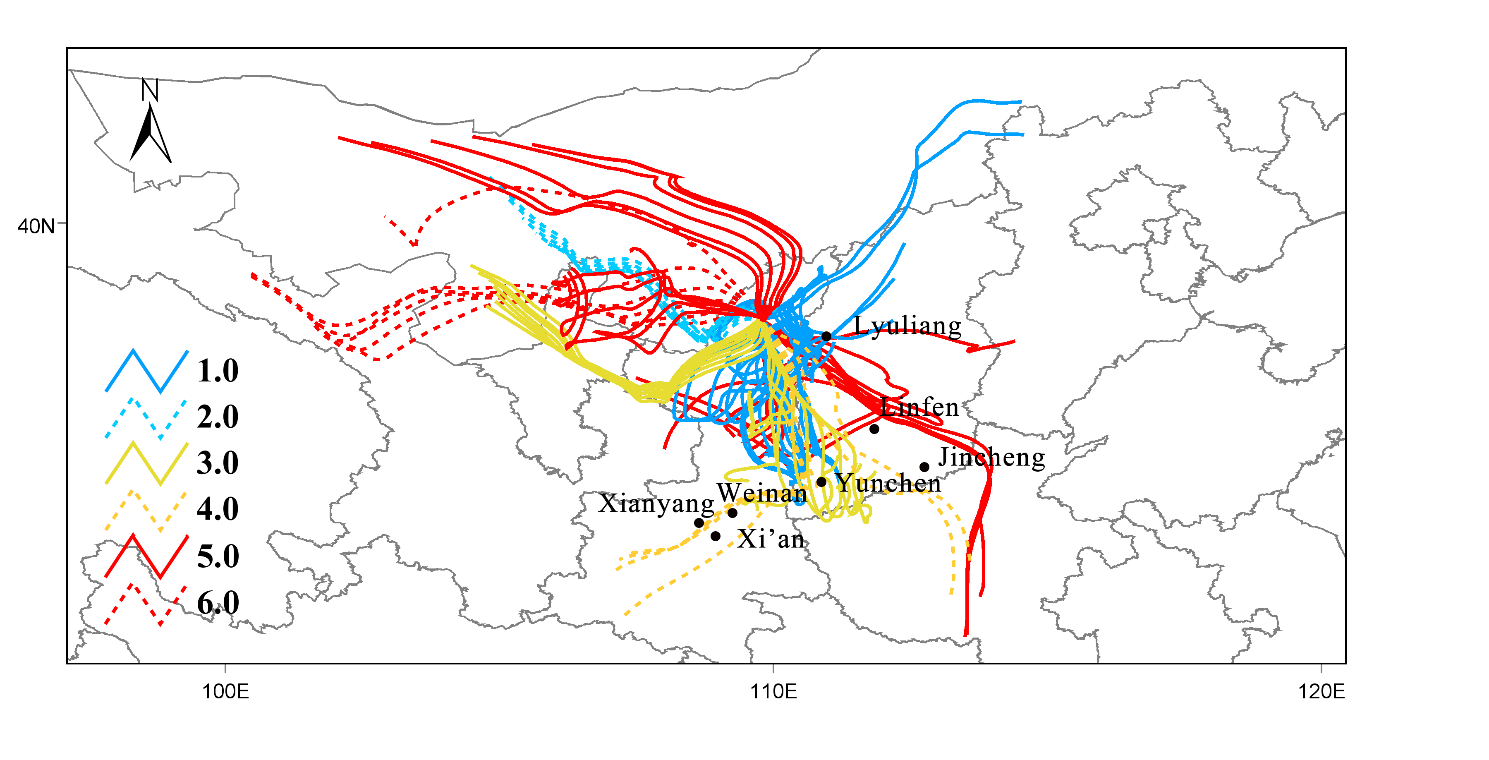


Figure S3 48h backward trajectories during six EPs.





Figure S4 The change of Qrobust/Qexpected in different change of factor’s number.


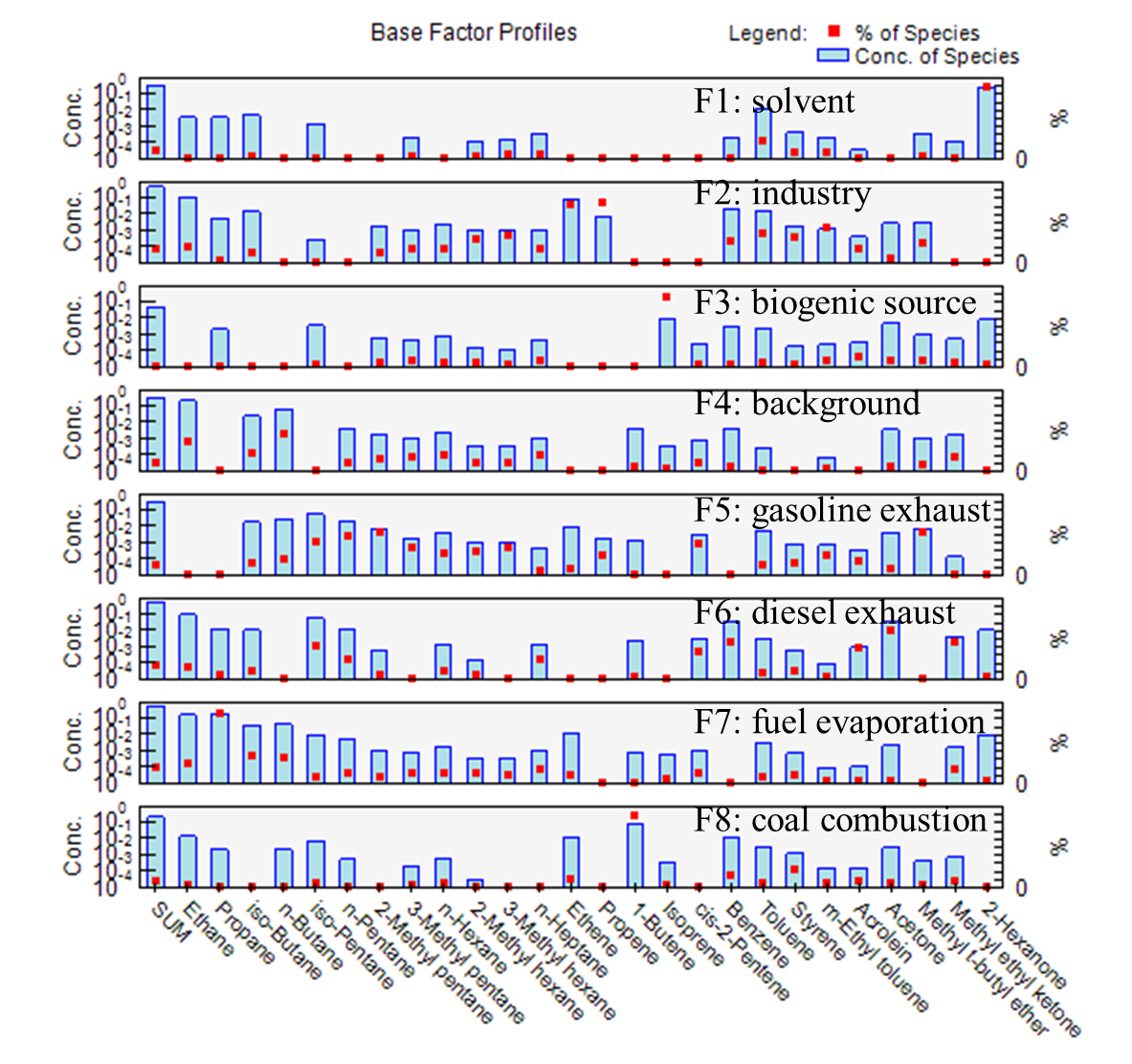


Figure S5 PMF source profile
